# Disjunction of conjoined twins: Cdk1, Cdh1 and separation of centrosomes

**DOI:** 10.1186/1747-1028-1-12

**Published:** 2006-06-22

**Authors:** Karen Crasta, Uttam Surana

**Affiliations:** 1 Institute of Molecular and Cell Biology, Proteos, 61 Biopolis Drive, 138673, Singapore

## Abstract

Accurate transmission of chromosomes from parent to progeny cell requires assembly of a bipolar spindle. Centrosomes (spindle pole body in yeast) are critical for the biogenesis of this complex mitotic apparatus since they confer bipolarity on the spindle and serve as the site of microtubule polymerization. In each division cycle, the centrosome is duplicated and the sister-centrosomes move away from each other, forming the two poles of the spindle. While the structure and the duplication of centrosomes have been investigated extensively, the understanding of the control of their segregation remains scant. Recent findings are beginning to yield insights into the regulation of centrosome segregation in yeast and its link to the mitotic kinase.

## Background

Segregation of chromosomes to the two opposite poles of a dividing cell is central to cellular reproduction. Fittingly, the mitotic spindle, which is primarily responsible for progressive separation of sister-chromatids during anaphase, is a tension-ridden, bipolar structure with centrosomes constituting the two poles. Centrosomes are also the microtubule organizing centers (MTOC) of a spindle [1]. In a typical metaphase spindle, the two centrosomes are in a 'face-to-face' configuration, separated by a set of overlapping microtubules, emanating from each centrosome towards the other (pole-to-pole microtubules) [2]. A second set of microtubules, called astral microtubules, radiate from each centrosome towards the cell cortex. A third set, termed kinetochore microtubules, emanate from the centrosomes and eventually attach to duplicated chromosomes at the kinetochores such that each sister kinetochore is linked to one pole that is directly facing it while the other is attached to the opposite pole. Due to the dynamic nature of the microtubules and the activities of the proteins associated with them (microtubule associated proteins or MAPS), the spindle poles (centrosomes) tend to move away from each other, causing the sister chromatids to be pulled in the opposite direction. In a metaphase spindle, this tendency is opposed by proteins known as cohesins that tether the sister chromatids together. Such opposing forces within the spindle are what make it a tension-ridden structure.

The centrosome is pivotal to the biogenesis of the mitotic spindle. In many animals, assembly of the first spindle in the fertilized egg is dependent on the MTOC (in the form of a basal body centriole) contributed by the sperm since oocyte centrosome degenerates sometime during oogenesis. Hence, during fertilization, the sperm contributes not only DNA but also MTOC for construction of a spindle [3]. The incoming centriole then recruits maternal components that constitute the pericentriolar material (PCM) [4]. Similarly, during division a cell inherits only one centrosome from its mother and has to build a complex spindle structure starting from this centrosome. In each cell cycle, centrosomes are duplicated and separated precisely to serve as two poles of the mitotic spindle, both acting as the organizing centers for nuclear and astral microtubules. It is quite astounding that a multi-protein structure like the centrosome is exactly duplicated without using a preexisting counterpart as a template, at least not in the sense that a template is used for copying DNA. We first take a brief look at this unique duplication process.

## Replicating the centrosome and spindle pole body

**Figure 1:**
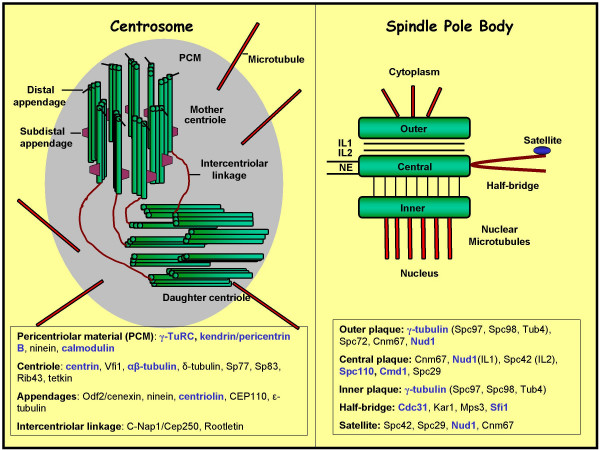
**Schematic representation of a Centrosome and Spindle Pole Body (SPB)**. Shown in boxes are the main components of the centrosome and SPB. Homologous components of the two structure are highlighted in blue.

**Figure 2:**
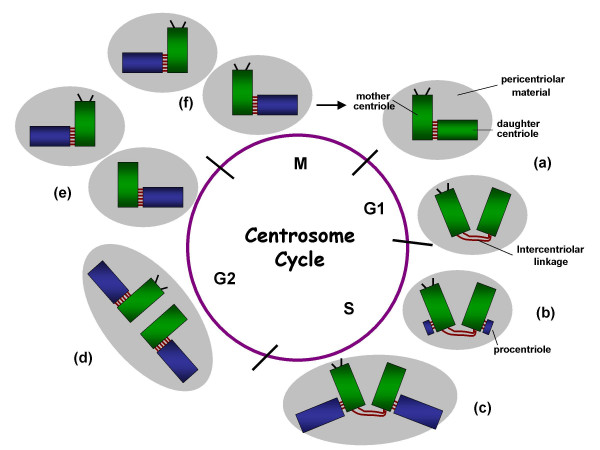
**The Centrosome Duplication Cycle**. The centrosome consists of mother and daughter centrioles (green), that are interconnected by an intercentriolar linkage (red) and are embedded in the pericentriolar material (grey) which anchors the microtubules. The mother centriole can be distinguished by the presence of appendages (black lines). (a) During G1, cells lose their orthogonal arrangement. (b) As G1/S, a procentriole (blue) forms perpendicular to each centriole. (c) During S phase, the new centrioles elongate. (d) At G2, the two newly formed centriole pairs disconnect, and (e) by G2/M, the PCM is also divided between the centrioles. (f) At the end of the cycle, the daughter centrioles acquire appendages and behave as a mother centriole during the subsequent cycle.

Like chromosomes, centrosomes are precisely duplicated, but only once in each cell cycle. In animal cells, each centrosome is composed of a pair of centrioles and the surrounding dense fibrillar mass known as the pericentriolar material (Figure 1). The centrioles in the pair are referred to as mother and daughter centrioles where the mother centriole can be distinguished by the presence of distal and sub-distal appendages. The centrioles themselves are cylindrical structures, each built from nine microtubule-triplets (doublet or singlets in some organisms) arranged in a 9-fold axis of symmetry and lie juxtaposed to each other such that their long axes are perpendicular to each other (also known as orthogonal arrangement) (Figure 1). Incidentally, centrioles are very similar in structure to basal bodies, the organelle located at the base of cilia [5]. In order to build two centrosomes from one, the pair of centrioles undergoes a duplication cycle (Figure 2). During G1 phase of the cell cycle, the centrioles lose their orthogonal arrangement in that the daughter centriole separate slightly from the mother centriole but remain tethered by a flexible connection. As cells enter S phase, a precursor centriole (procentriole) appears perpendicular to the proximal end of each of the parental centriole and continues to elongate through S phase to attain the same length as the parental ones. At G2/M, the pairs of centrioles disconnect completely, along with the divided pericentriolar material, to form two separate (mother and daughter) centrosomes. This is followed by maturation of the immature daughter centriole by full acquisition of appendages that contain proteins such as cenexin, ninein, CEP110 or ε-tubulin [6-8]. It is interesting to note that like DNA replication, the duplication of centrioles is a semi-conservative process in that each pair of centrioles consists of one old and one new member. That centriole duplication is semi-conservative but nucleus-independent has given support to the idea that centrosomes contain their own nucleic acids, like mitochondria and chloroplasts do. Indeed, presence of RNA in surf clam oocyte has been reported [9]; however the role it might play in centriole duplication is far from clear.

Some of the paradigms of centrosome duplication have been based on the exquisite work done on the spindle pole bodies (SPB), the centrosome-equivalent in yeast [10]. At the structural level, SPBs do not bear much resemblance to centrosomes. In budding yeast *Saccharomyces cerevisiae *SPB is a cylindrical structure embedded in the nuclear envelope and comprises three distinct layers (Figure 1). The outer plaque faces the cytoplasm, contains proteins such as Tub4, Spc98, Spc97 and Spc72 and nucleates astral microtubules [11]. The inner plaque faces the nucleoplasm and extends the nuclear microtubules that consist of both pole-pole microtubules and the kinetochore microtubules. The central plaque is embedded in the nuclear envelope and anchors both outer and inner plaques. Proteins such as Spc29, Spc42 and Cmd1 have been localized to this layer. The central plaque also contains an electron-dense structure known as the 'half-bridge'. The half-bridge is an important appendage because its distal end mediates the assembly of a new SPB. A number of proteins including Cdc31 (centrin homolog), Kar1 and Sfi1 (similar to human Sfi1) and Mps3 have been localized to this structure. The crystal structure of Sfi1-centrin filaments suggests that repeats in Sfi1 form a continuous filament to which centrin binds. Hence, multiple interactions between Sfi1 and centrins is proposed to gives the half-bridge its characteristic shape [12].

**Figure 3:**
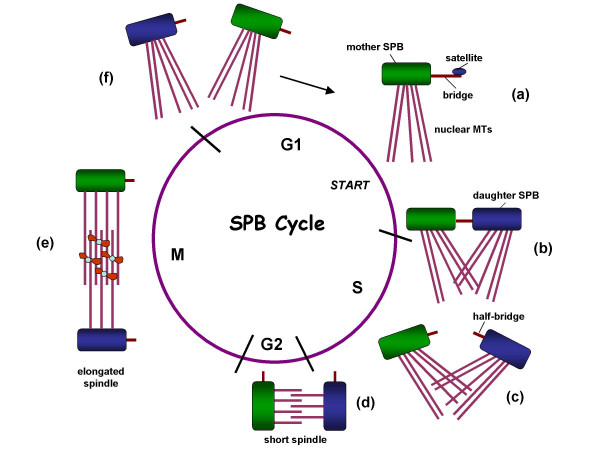
**The Spindle Pole Body (SPB) Duplication Cycle**. (a) During G1 (prior to START), the distal end of the half-bridge (red) of the mother SPB (green) acquires a satellite (blue oval). (b) As cells traverse START, the satellite enlarges to form the daughter SPB (blue rectangle). (c) At late S phase, the bridge is severed and (d) the SPBs move apart to form a short spindle. (e) During mitosis, the spindle poles move further apart as the spindle elongates. (f) At the end of the cycle after spindle disassembly, each cell acquires a SPB.

The duplication of SPBs, like that of centrosomes, is an intricate affair. At the time of its birth, the yeast daughter inherits from its mother one SPB bearing a half-bridge. During G1, the precursor for a new SPB, called satellite, is assembled on the cytoplasmic side of the half-bridge's distal tip (Figure 3) and requires self-assembly of Spc42 [13]. As cells traverse START, the satellite expands to form a duplication plaque whose structure resembles the cytoplasmic side of the mature SPB. During this time, the half-bridge also extends under the duplication plaque and forms a complete bridge by fusion of its cytoplasmic and nuclear fronts. The bridge then retracts to some extent allowing the insertion of duplication plaque into the nuclear membrane and the assembly of the nucleoplasmic side. Finally, the old and the new SPBs lie in a side-by-side configuration, interconnected by the bridge. In late S phase, the bridge is severed which allows the two SPBs to segregate away until they come face-to-face with each other, separated by an array of interdigitated microtubules. This structure is what is generally known as the short spindle.

Centrosome/SPB duplication and its integration with other cell cycle events involve a host of structural and regulatory proteins. We will not discuss these components individually or the details of the functional interactions among them and how they contribute to the various stages of duplication process since a number of excellent reviews on this subject are already available in the existing literature [11,14-17]. Instead, we will focus our attention on an event that occurs after duplication i.e. centrosome separation, without which a spindle cannot be assembled in its characteristic bipolar form even though cells can duplicate centrosomes successfully.

## Centrosome separation

While centrosome duplication has been fairly well documented, the separation of duplicated centrosomes is poorly understood. In the case of vertebrate cells, the mother and daughter centrioles (and the duplicated centrosomes) appear to be tethered since centrioles remain paired in centrosome preparations. Electron microscopy studies show an electron dense material between the mother and daughter centrioles in isolated centrosomes. However, the molecular nature of this linker (intercentriolar linkage) remains unclear. (It should be noted that the linkage between the mother and daughter centrioles is what later, after duplication, becomes the linkage between the two pairs of centrioles i.e. centrosomes). While *in vivo *existence of a linker is not yet unequivocally proven, some of the proteins that regulate centrosome separation (or centriole disjunction) have been identified. Nek2A, a NIMA related kinase [18], forms a complex with the catalytic subunit of phosphatase PP1 and C-Nap1, a large coiled-coil protein (280 kDa) thought to provide a docking site for the linker. Nek2A is able to phosphorylate itself and C-Nap1, whereas PP1 can dephosphorylate both Nek2A and C-Nap1. It has been reported that inhibition of C-Nap1 activity or over-expression of Nek2A leads to premature separation of mother and daughter centrioles. Moreover, while co-expression of PP1 and Nek2A can prevent centriole separation, inhibition of PP1 activity promotes the separation. These observations suggest that mutual regulation of the C-Nap1, PP1 and Nek2A is important for the control of centrosome cohesion. Recently, a C-Nap1 interacting protein, called Rootletin, has been shown to participate in centriolar cohesion [19]. It is also phosphorylated by Nek2 and is removed from centrosomes at the onset of mitosis. Like in the case of C-Nap1, inhibition of Rootletin results in centrosome splitting.

Motor proteins are known to play an essential role in bipolar spindle assembly [20]. Eg5, a plus-end directed homotetrameric motor protein belonging to BimC (Kinesin-5) family is of particular importance in this context [21,22]. It can both push apart pole-to-pole microtubules as well as recruit microtubules into bundles [23]. Inhibition of Eg5 by a small molecule inhibitor, monastrol, prevents centrosome separation and leads to monopolar spindles suggesting that its functions are essential for biogenesis of a bipolar spindle [24]. The association of Eg5 with spindle apparatus is regulated via phosphorylation by Cdk1 in human cells [25], suggesting a direct involvement of Cdk1 in centrosome separation. In addition to Cdk1, polo and aurora-A kinases are also implicated in centrosome separation. In both Xenopus and Drosophila, loss of aurora-A kinase activity results in a failure to separate centrosomes [26,27]. In both Drosophila and human cells, centrosomes fail to separate in the absence of polo kinase [28,29]. At least in vertebrate cells, it is possible that polo and aurora-A kinases may contribute to complete removal of C-Nap1 from centrosomes [30].

## Separation of yeast SPBs until now

By the time budding yeast cells enter S phase, the SPBs are already duplicated and are linked by a complete bridge. At the end of S phase, the bridge is severed and the SPBs move away from each other, each carrying a half bridge, to constitute two poles of a short spindle. A number of cellular conditions can result in failure to separate SPBs and assembly of a bipolar spindle: 1) simultaneous deficiency of plus-end directed kinesin motors Cin8 (homolog of vertebrate Eg5) and Kip1 [31,32], 2) deficiency of B-type cyclins Clb1, Clb2, Clb3 and Clb4 [33], 3) inability to dephosphorylate the conserved tyrosine 19 on Cdk1 (Cdc28) [34], and 4) over-expression of tyrosine kinase Swe1 (budding yeast homolog of Wee1) [34]. These observations suggest that, like centrosomes, SPB separation also require microtubule-associated proteins (MAPs) such as Cin8 and Kip1, and Cdk1 activity. Although no additional proteins seem to have been clearly implicated in SPB separation, it should be noted that the inability to separate SPBs due to combined deficiency of Cin8 and Kip1 can be partially suppressed by deletion of a minus end-directed motor Kar3 [35]. This has prompted the idea that SPB separation requires interplay of opposing forces and that additional regulators involved. Such antagonistic forces also profoundly influence the overall spindle dynamics that presumably influences the elongation and spindle length during mitosis. While the requirement for kinesin motor proteins Cin8 and Kip1 could be tentatively explained in terms of the need to move SPBs apart, the role of Cdk1 (and specifically its activation by Tyr 19 dephosphorylation) had not been elucidated.

## MAPs, Cdh1, Cdk1 and SPB segregation

**Figure 4:**
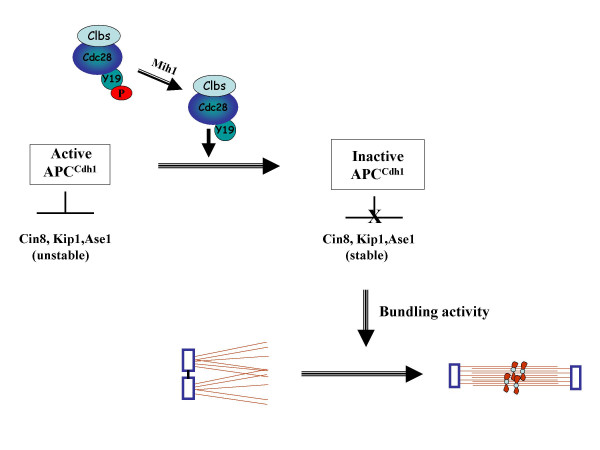
**Model depicting role of activated Cdc28 (Cdk1) in SPB separation in budding yeast**. Tyrosine dephosphorylation of Cdc28 by Mih1 leads to inactivation of Cdh1 resulting in stabilization of microtubule-associated proteins Cin8, Kip1 and Ase1 whose bundling activity catalyzes SPB separation and formation of the bipolar mitotic spindle.

Recent findings functionally connect the activities of MAPs, Cdh1 (an activator of the ubiquitin ligase called anaphase promoter complex or APC) and Cdk1 (Cdc28) to SPB separation [36]. This study analyzed two mutants each carrying a different version of Cdc28: 1) *cdc28Y19E*, an allele in which evolutionarily conserved tyrosine 19 is replaced by glutamic acid, thus mimicking a constitutively phosphorylated state and 2) *cdc28-as1*, an allele with F88G substitution that alters ATP binding site and is sensitive to a bulky ATP analogue 1NM-PP1 [37]. At their respective restrictive conditions, both mutants arrest in G2/M, with 2N DNA content and duplicated but unseparated SPBs, and hence fail to assemble a bipolar spindle. In both mutants the levels of microtubule associated proteins Cin8, Kip1 and Ase1 were found to be very low. Pulse-chase experiments showed that these proteins are highly unstable in the mutant cells. Deletion of Cdh1 not only restored the levels of these MAPs but also suppressed the inability to separate SPBs and allowed formation of a bipolar spindle. While SPB separation and spindle formation were also restored to a large extent by expression of proteolysis-resistant version of any one of the MAPs, over-expression of Cdh1 reduced the levels of the MAPs and completely precluded spindle formation. These observations strongly suggest that in the absence of mitotically active Cdc28, Cdh1 remains active in cells and targets Cin8, Kip1, Ase1 for proteolysis, implying that activated Cdc28/Clb kinase (by Tyr 19 dephosphorylation) is responsible for inactivating Cdh1, allowing accumulation of these proteins to induce SPB separation. Consistent with this, Cdh1 is hypo-phosphorylated and Cin8 is highly ubiquitylated in *cdc28 *mutant cells. This conclusion is supported by a strong correlation between the dephosphorylation of Tyr 19 residue of Cdc28, accumulation of Cin8, Kip1, Ase1 and separation of SPBs during a normal division cycle (Figure 4). The finding that ectopic expression of proteolytic-resistant Cin8, Kip1 or Ase1 is sufficient to catalyze SPB separation in cells devoid of Cdc28-Clb activity argues that stabilization of MAPs is perhaps the predominant role of Cdk1 in the separation of SPBs.

## Bundling, motoring and bridge-breaking

That motor proteins are required for separating SPBs seems intuitively obvious since the act of separation needs physical movement. Cin8, Kip1 and Ase1 can each induce SPB separation in *cdc28 *mutant cells [36]. However, Ase1 does not exhibit any motor activity. What property, then, do these proteins share that allows SPB separation? The ability to recruit microtubules into bundles (bundling activity) is common to these three proteins. In the case of Cin8, bundling and motor activity can be separated by mutation: of certain amino acid residues: while R196K substitution eliminates the motor activity, R394A, H396A and E871A collectively abolish the bundling activity [38]. Interestingly, ectopic expression of mutant *cin8*, defective in motor activity but proficient in bundling activity, is able to induce SPB separation and spindle formation in *cdc28 *mutants described above but bundling-defective mutant cin8 is unable to do so [36]. Hence, while motor activity of Cin8 is dispensable for SPB separation, bundling activity is not (Figure 4). One implication of this conclusion is that the act of bundling, which involves conversion of interdigitating microtubules emanating from each SPB into an anti-parallel array of microtubules, generates enough shear force to break the bridge connecting the two SPBs.

It can be argued then, that the physical movement required for converting side-by-side SPBs into a short spindle is caused by the shearing force generated by bundling but not motor activity. Later in the cell cycle, when the short spindle extends into a long anaphase B spindle, motor activity plays a predominant role. Thus, two distinct activities of motor proteins like Cin8 can be considered important for two different stages of spindle biogenesis. It remains to be seen if bundling and motor activities are regulated differently during the cell cycle.

## Centrosomes versus SPBs

Despite little structural resemblance between centrosomes and SPBs, some broad parallels in the process of their duplication and separation can be seen. Both are multi-protein assemblies that cells must duplicate every division cycle, by using the preexisting mother structure, not as a template for copying but as a surface for seeding the assembly of a new one; in the case of centrosomes it is the proximal end of the mother centriole, whereas for the SPBs it is the distal end of the half-bridge that acts as a platform for seeding. As the SPB half-bridge lengthens and partially retracts during the duplication cycle, the fibrous material that connects the proximal ends of mother and daughter centrioles also shows lengthening and shortening during duplication. Human homologs of some of the proteins involved in SPB biogenesis, such as Cdc31 (centrin HsCen1, HsCen2, HsCen3), Sfi1 (HSfi1), Cmd1 (calmodulin Calm1, Calm2, Calm3) and Spc110 (kendrin) have also been found. Moreover, SPB biogenesis in yeast is drastically affected by over-expression of human centrin 3. Such parallels suggest that there may be a common building strategy that the construction of both centrosome and SPB follows [10].

Duplication of centrosomes and SPBs is followed by their separation; in both cases, it involves plus-end-directed kinesin motors and Cdk1 activity. As mentioned earlier, for centrosome separation (disjunction), Nek2A mediated displacement of C-Nap1 and the activity of Eg5 appear to be necessary. The involvement of Cdk1 is through phosphorylation of Eg5 which allows its binding to centrosomes. Unlike SPB separation in yeast where Cdk1's role is to stabilize Cin8, Cdk1-mediated stabilization of Eg5 has not been reported. However, Eg5 contains a KEN-box [39] which makes it a potential substrate for APC^Cdh1^. Moreover, over-expression of Cdh1 prevents centrosome separation in human cells [40] suggesting a possible involvement of Cdh1 and Cdk1-mediated stabilization of Eg5 in centrosome separation. As for the requirement of motor versus bundling activity, recent findings clearly suggest that motor activity of Cin8 is dispensable for SPB separation while microtubule binding and bundling activities are essential [36]. It has been reported that in *Xenopus *egg extracts, mutant version of Eg5 with ~6 fold reduced motor activity can fairly efficiently assemble bipolar spindles though the assembly process is slower suggesting that the motor activity of Eg5 may be dispensable. However, the importance of Eg5's bundling activity in centrosome separation is not known [22].

A number of key issues connected with centrosome duplication/separation such as the control of centrosome numbers, relationship between centrosome biogenesis and genomic instability, the nature of the seeding process that initiates duplication, details of the motor protein-mediated separation, identification of additional regulators and their roles to name a few, need to be resolved. Nonetheless, centrosomes/SPBs and the spindle they help to assemble, remain among the most beautifully symmetric cellular structures and still pose a challenge to be understood in detail.
